# Travelling Editorials

**Published:** 1843-03

**Authors:** 


### THE WESTERN JOURNAL.

Vol. VII.—No. III.

LOUISVILLE, MARCH 1, 1843.

### TRAVELLING EDITORIALS.

This phrase admits of no less than three applications. It may mean editorials for the benefit of travellers; editorials of such excellence and interest, that they will travel far and wide among the profession; or editorials written while travelling. It is in the last sense that we use the expression. Of the course and limits of the journeyings on which we have entered, an intimation was given in our January number. Of the *materiel* which we may work up into our monthly communications, we cannot speak so definitely; inasmuch as we know not what may offer, and do not intend to be rigidly professional. That would be to come with "malice prepense" into conflict with the tastes and habits of our brethren, many of whom, with ourselves, know a good deal out of the profession, however little they and we may know in it. When Lord Brougham was made Chancellor of England, a London wag exclaimed, what a pity it is, that his lordship does not know something of the duties of his high office, for he would then be a man of universal learning. Disclaiming all personal application of this sarcasm, we shall proceed to state, in this first letter, the object which is *carrying* us to the shores of the Gulf.

### *Diseases of the West.*

More than twenty years ago we announced the design of publishing a work on this subject. Of the causes which have delayed its preparation, we shall not speak in detail, and will mention one only. Reflection soon convinced us, that the undertaking was of greater magnitude and difficulty than it first appeared, and could not, indeed, be accomplished without extensive and patient personal observation, particularly in the the north and the south, that the *feathering* out of our endemics in those opposite directions might be distinctly ascertained. But excursions for that purpose were impracticable, till the last summer, when, in visiting the shores of the Lakes, we commenccd the series of medical travels which have now been resumed. In their prosecution, it is our object, 1st. to acquire a knowledge of the modifications of our climate, from the Lakes to the Gulf, with their influence on the constitutions of the people. 2d. To note the various geological and topographical conditions, which may be supposed, directly or indirectly, to occasion or prevent diseases. 3d. To observe the diet, drinks, occupations, and manners and customs of the inhabitants, as predisposing to, producing, or preventing diseases. 4th. To obtain from medical gentlemen, in all parts of the country, such information concerning the diseases prevalent in their respective localities, as can be drawn from them by personal interviews. 5th. To collect facts for a comparative estimate of the physiology and pathology of the Europo-American, the Indian and the Negro.

Our field of observation extends from Michigan to Florida, and from the western slopes of the Allegheny mountains, to Missouri, Arkansas, and Iowa.

Such is the enterprize on which we have entered at an advanced period of life, though with some of the activity and feeling which belong to earlier years. Should we not live, or otherwise fail, to achieve it, we have the satisfaction to believe that our researches may still be of some benefit to the profession, inasmuch as we can scarcely fail to record many valuable facts, which might otherwise be lost, and which by some abler hands may be presented to the public.

In addressing these paragraphs to our readers, and all other medical gentlemen within the extended region we have designated, we are actuated by an earnest desire to secure their co-operation. Without it, a failure is inevitable; with it, *some* degree of success may be regarded as almost certain. Therefore we respectfully solicit their co-operation. We shall devote the months of March and April to Florida, South-Alabama, and the south-east of Mississippi; May to the Delta of the Mississippi; and June to the upper parts of Louisiana, the western portion of Mississippi, and the eastern part of Arkansas; and we take this method to request such of our readers as reside in those States, to prepare for us, in writing, such transcripts of their experience as may be fit materials for our projected work. We would, moreover, extend this request to all who practise medicine in the various States west of the Alleghenies, in the hope, that they will at once perceive the necessity of their co-operation, and that they will forward to us, at no distant time, the facts which are requisite to a full history of our most important diseases.

*Bed and Banks of the Mississippi.*

If it had a rocky bed and banks, well defined, and unchangeable, the Mississippi would be the most magnificent of rivers. As it is, neither beauty nor grandeur can be ascribed to the "Father of Waters." His great attribute is power, but in its exercise he is so often obstructed and foiled as to detract greatly from his dignity. At the very moment of writing this sentence, the afternoon sun shines upon us through the stern windows of the Queen of the West, showing that she runs nearly north-east instead of south. Thus it is that the tide of rolling waters is deflected from its course, and almost turned back, by the banks of mud and sand which itself has brought down and deposited. Struggling to reach the Ocean, the *true* father of rivers, it is compelled to fight its way, to turn aside, to abandon its old channels and excavate new ones. For a short distance the waters may flow between parallel and equally elevated banks, but they soon spread out on one side, or divide into different channels, which, ere long, they are compelled by new accumulations of sand and sunken trees to abandon. Seen from the surface of the stream, the higher banks present a perpendicular and ragged front, from which masses are perpetually falling into the stream, to pollute its waters for a time, and then desert them for a new location. The horizontal and waved stratification of these banks, mark them as deposites from the river itself, consisting of varying proportions of sand and clay, with enough of the carbonate of lime to render many specimens slightly effervescent. A medium height of water is that which does greatest mischief to the banks; and its abrading force is greatly increased by the waves raised by a southerly wind, and by the wheels of the numerous and powerful steamers, which, in ascending, overcome, in descending, outstrip the current. As the latter must long continue to be an *increasing* cause, the work of destruction is not likely to abate. Opposed to the perpendicular and crumbling banks, are low and shapeless beaches, which in high water are buried up, but in low, present extensive deposites of drift on their slopes, while their flatter surfaces are covered with dense groves of young cotton trees. Now and then three, very often two ranks of this kind, having different ages, may be seen from the river, the youngest in front, the oldest in the back ground. This tree, the *Populus angulata* of the botanists, gives character to the SYLVA MISSISSIPPIENSIS, and, if not the offspring, is at least the adopted child of the waters, which deposite the soil in which it attains a magnitude unknown elsewhere; while its down enveloped seeds, in countless millions, are transported by the current to every new plantation, where they speedily germinate. The trunk of this tree makes excellent fuel for steam-boats, and thus will the Mississippi forever create the means by which man will stem its mad and mighty current. If the trees upon its banks were to perish, or the cotton wood become incombustible, the effect would be felt throughout the entire west.

### *Tertiary Formations.*

Such of our up country readers, as have trodden from their infancy upon rocks of the secondary class, would do well, should they descend the river, to keep a good look out for the Chickasaw bluffs of Randolph and Memphis. Vicksburg, Grand-Gulf, and Natchez, present fine sectional views of the tertiary formations which stretch so extensively through West-Tennessee and the middle portions of Mississippi and Alabama.

### *Steamboat Bars.*

Now, that ardent spirits are no more seen on the dinner tables of our steamers, and the number of passengers, who desire or dare to frequent the bars, is greatly reduced, it would seem to be natural, and not difficult, to suppress them entirely. The chief motive for retaining them is destroyed, when they yield but little profit; and, therefore, the owners of boats might be expected not to resist their abolition. But under what influence can this important reform be brought about? We answer, that of the friends of temperance, exerted on the general government. After having legislated on the registration and inspection of steamboats, even to a prescription for tiller chains, it would be no unwarrantable exercise of power, to suppress the sale of intoxicating drinks, both to passengers and operatives. The principle on which Congress might place this interposition, is the same as that on which they have directed chains for ropes—the safety of passengers. No observing man can believe but that many of the disasters which have happened to our steamboats were the effect of intemperance, particularly indulged in at night; and that, with *its* suppression, the number of accidents would be signally abated. We cannot but ardently desire to see the friends of temperance move in this matter.

*India Rubber Life-Preservers.*

On our present voyage down the Ohio and Mississippi, not less than before, we have marvelled to see so few of these valuable articles. Hundreds who now travel on steamers without them, would, if they had never been invented, deplore the want which they now neglect to supply. With a good life preserver around her chest, even the most helpless and timid woman, would be safe for several hours unless the water might chance to be as, it now is, but two or three degrees above the freezing point; and still but few ladies are provided with them, and still fewer gentlemen, although none of the former and not many of the latter know how to swim. By the way, it is a serious and absurd defect, in the physical education of our daughters, that they are not taught to swim; as it is a valuable exercise in early life, and a source of confidence and security even to old age. In past times, when our women remained at home, because they could not travel, the latter consideration was of less moment than at present, when so many perform long voyages, at every moment exposed to casualties which might drown them, merely because a day had not been devoted to their instruction in the art of swimming.

### *Steamboat Explosions.*

All passengers who are exposed to these accidents ought to know, that the steam which spreads through the cabin, when explosions occur, will not scald those parts of the body which are covered even thinly. Thus, those, who are in their berths when such an accident happens, should lie still, and cover up their heads, instead of rising, as has so often happened; and those who are up, might protect themselves by covering their hands and face with an apron, the skirts of a coat, or even a silk handkerchief. Reaching the skin through such a fabric, steam, which would otherwise blister, will scarcely redden it. A further precaution, not unworthy of notice, is to suspend or hold the breath, at the moment of becoming enveloped in the steam, by which its introduction into the larynx and lungs is prevented.

### *Winter Temperature of the Mississippi.*

From the mouth of the Ohio to New Orleans, the distance is one thousand fifty-nine miles—the distance on the meridian 3 degrees, rejecting small fractions. Thus in passing through a degree of latitude, the river flows three hundred and fifty-three miles. Its temperature at the junction between it and the Ohio, we found to be 34°, which rose at a constant rate to New Orleans, where we find it 42°; giving an increment of two degrees and two thirds of surface temperature, for one degree of latitude, and for every three hundred and fifty-three miles of current.

### *Climate of New Orleans.*

The very night of our arrival afforded conclusive evidence that great and sudden changes of temperature occur in the delta of the Mississippi. On the 14th of February, when below Baton Rouge, the heat of the air, at half past 11 A. M., was 71°. Rain with thunder and lightning ensued, accompanied by westerly winds, and in two hours the thermometer sunk 19°. Before sun-rise the next morning, at the wharf, it was at 28°, making a fall of 43° in twenty hours. A more extreme change seldom occurs in any latitude. The next morning it was at 32°. This morning at 34°, with a copious white frost. The liability of this climate to such great depressions of temperature, so late as the middle of February, should admonish those who seek for relief from pulmonary diseases, by a voyage to the south, to look out for disappointment, if they start in winter, or stop here. D.

New Orleans, February 17, 1843.

